# Carvacrol Suppresses Inflammatory Biomarkers Production by Lipoteichoic Acid- and Peptidoglycan-Stimulated Human Tonsil Epithelial Cells

**DOI:** 10.3390/nu14030503

**Published:** 2022-01-24

**Authors:** Niluni M. Wijesundara, Song F. Lee, Ross Davidson, Zhenyu Cheng, H. P. Vasantha Rupasinghe

**Affiliations:** 1 Department of Biology, Faculty of Science, Dalhousie University, Halifax, NS B3H 4R2, Canada; niluniw@dal.ca; 2 Department of Plant, Food, and Environmental Sciences, Faculty of Agriculture, Dalhousie University, Truro, NS B2N 5E3, Canada; 3 Department of Animal Science, Faculty of Animal Science and Export Agriculture, Uva Wellassa University, Badulla 90000, Sri Lanka; 4 Department of Microbiology & Immunology, Dalhousie University, Halifax, NS B3H 6R8, Canada; song.lee@dal.ca (S.F.L.); Ross.Davidson@nshealth.ca (R.D.); zhenyu.cheng@dal.ca (Z.C.); 5 Department of Applied Oral Sciences, Faculty of Dentistry, Dalhousie University, Halifax, NS B3H 4R2, Canada; 6 Canadian Center for Vaccinology, Dalhousie University, Nova Scotia Health Authority, and the Izaak Walton Killam Health Centre, Halifax, NS B3H 4R2, Canada; 7 Department of Pathology, Faculty of Medicine, Dalhousie University, Halifax, NS B3H 4R2, Canada; 8 Department of Pathology and Laboratory Medicine, Division of Microbiology, Queen Elizabeth II Health Sciences Centre, Nova Scotia Health Authority, Halifax, NS B3H 1V8, Canada

**Keywords:** anti-inflammatory, carvacrol, streptococcal pharyngitis, cytokines, LTA, PGN, inflammation, *Streptococcus pyogenes*

## Abstract

Pharyngitis is an inflammation of the pharynx caused by viral, bacterial, or non-infectious factors. In the present study, the anti-inflammatory efficacy of carvacrol was assessed using an in vitro model of streptococcal pharyngitis using human tonsil epithelial cells (HTonEpiCs) induced with *Streptococcus pyogenes* cell wall antigens. HTonEpiCs were stimulated by a mixture of lipoteichoic acid (LTA) and peptidoglycan (PGN) for 4 h followed by exposure to carvacrol for 20 h. Following exposure, interleukin (IL)-6, IL-8, human beta defensin-2 (HBD-2), epithelial-derived neutrophil-activating protein-78 (ENA-78), granulocyte chemotactic protein-2 (GCP-2), cyclooxygenase-2 (COX-2), tumor necrosis factor-alpha (TNF-α), and prostaglandin (PGE_2_) were measured by enzyme-linked immunosorbent assays (ELISA). The levels of pro-inflammatory cytokines, IL-6, IL-8, ENA-78, and GCP-2 were decreased in a carvacrol dose-dependent manner. The production of HBD-2 was significantly suppressed over 24 h carvacrol treatments. PGE_2_ and COX-2 levels in the cell suspensions were affected by carvacrol treatment. TNF-α was not detected. The cell viability of all the tested carvacrol concentrations was greater than 80%, with no morphological changes. The results suggest that carvacrol has anti-inflammatory properties, and carvacrol needs to be further assessed for potential clinical or healthcare applications to manage the pain associated with streptococcal pharyngitis.

## 1. Introduction

Inflammation is a natural defense mechanism of the body designed to eliminate harmful stimuli, including exogenous pathogens, and initiate the healing process through various chemical mediators and signaling pathways [1]. Pharyngitis is an inflammatory process of the posterior oropharynx [2]. Pharyngitis is one of the most common syndromes of upper respiratory tract (URT) infections. Although pharyngitis is usually self-limiting and a minor health concern, it is one of the leading causes of physician visits worldwide.

Bacterial pharyngitis is primarily caused by *Streptococcus pyogenes,* which is also known as group A *Streptococcus* (GAS). In North America, adults account for 5–15% of cases of streptococcal pharyngitis, while children account for 15–35% of the cases [3]. *S. pyogenes* first colonizes the tonsil epithelial cells, and these cells stimulate an innate immune response [4]. The tonsillar epithelial cells recognize *S. pyogenes* cell wall antigens through Toll-like receptors (TLR), such as TLR-2. These receptors identify pathogen-associated molecular patterns, including lipoteichoic acid (LTA) and peptidoglycan (PGN) [5,6]. Upon TLR recognition, tonsil epithelial cells produce pro-inflammatory cytokines, chemokines, prostaglandins, and tumor necrosis factor-alpha (TNF-α) [5,7]. Several inflammatory and infectious complications are associated with GAS pharyngitis, such as tonsillar hypertrophy, scarlet fever, and peritonsillar abscesses. In addition, post-infectious sequelae, such as acute rheumatic fever and post-streptococcal glomerulonephritis, have also been described.

Non-steroidal anti-inflammatory drugs (NSAIDs) such as ibuprofen, aspirin, ketorolac, diclofenac, and nimesulide have been widely recommended to manage acute inflammatory conditions [8], including streptococcal pharyngitis. It was found that cyclooxygenase (COX)-1 and -2, enzymes responsible for the synthesis of pro-inflammatory prostaglandins E_2_ (PGE_2_), are targets of NSAID [7].

The pharmaceutical and nutraceutical industry has a growing interest in novel sources of anti-microbial and anti-inflammatory agents. Bioactive compounds from medicinal plants have been explored as a source of potential to develop novel autoimmune-modulating, analgesic, and anti-inflammatory products. For example, we have previously shown that the ethanol and aqueous extracts of several herbal plants possess anti-inflammatory activities, decreasing the production of tonsil cell-associated inflammatory mediators, including cytokines, such as interleukin (IL)-8, human beta defensin-2 (HBD-2), granulocyte chemotactic protein-2 (GCP-2), and epithelial-derived neutrophil-activating protein-78 (ENA-78) [9].

**Figure 1:**
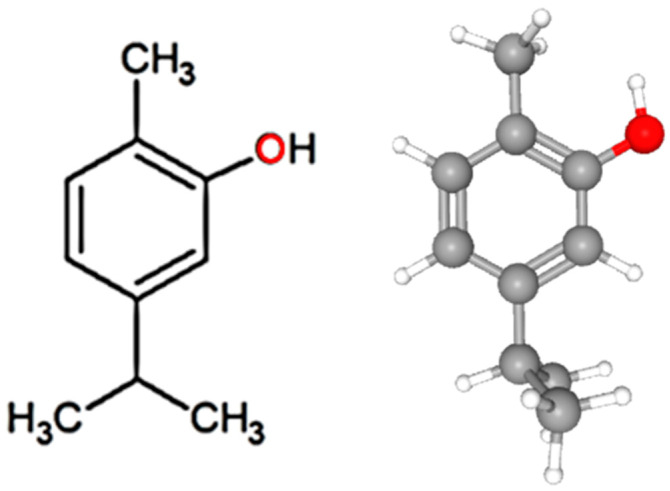
The chemical structure of carvacrol.

Carvacrol (5-isopropyl-2-methyl phenol, Figure 1) is a small monoterpene phenolic compound naturally found in the essential oils rich in the plants of the Lamiaceae family including *Oregano* L., *Thymus* L., and *Salvia* L. [10,11]. Several studies have reported on the anti-inflammatory [12,13,14] and analgesic [14] properties of carvacrol using different infectious inflammatory cell/animal models. Furthermore, the potential mechanisms of carvacrol were reported as inhibiting the synthesis of TNF-α, PGE_2_, and nitric oxide [12,15,16] and decreasing the levels of cytokines and COX-2 [13,16]. However, the effect of carvacrol on *S. pyogenes* antigen-induced tonsil epithelial cells and its inflammatory mediator production has not been reported. Therefore, the present study investigated the impact of carvacrol on cytokine production by the tonsil epithelial cells (HTonEpiCs) following LTA and PGN stimulation.

## 2. Materials and Methods

### 2.1. Chemicals and Reagents

Carvacrol (≥98%, food-grade), Dulbecco’s phosphate-buffered saline (DPBS), dimethyl sulfoxide (DMSO), lipoteichoic acid (LTA), 3-(4,5-dimethylthiazol-2-yl)-5-(3-carboxymethoxyphenyl)-2-(4-sulfophenyl)-2H-tetrazolium (MTS), and phenazine methosulfate (PMS) were obtained from Sigma-Aldrich (Oakville, ON, Canada). Peptidoglycan (PGN) was obtained from Cedarlane Laboratories (Burlington, ON, Canada). Human tonsil epithelial cells (HTonEpiCs), tonsil epithelial cell medium, tonsil epithelium cell growth supplement, poly-L-lysine (PLL), trypsin neutralization solution (TNS), and trypsin-ethylenediamine tetra acetic acid (0.25%) solution (TE) were purchased from ScienCell Research Laboratory (San Diego, CA, USA). Fetal bovine serum (FBS) was purchased from American Type Culture Collection (ATCC) (Manassas, VA, USA). 7-Aminoactinomycin D (7-AAD) stain was purchased from BioLegend, Inc. (San Diego, CA, USA). Enzyme-linked immunosorbent assay (ELISA) kits were purchased from different manufacturers: IL-6 and TNF-α ELISA kits were purchased from BD Biosciences (Mississauga, ON, Canada); human ENA-78, GCP-2, and human COX-2 ELISA kits were purchased from RayBiotech, Inc. (Norcross, GA, USA); a human BD-2 ELISA kit was purchased from PromoCell GmbH (Sickingenstraße, Heidelberg, Germany); and PGE_2_ and IL-8 kits were purchased from Invitrogen (Nepean, ON, Canada).

### 2.2. HTonEpiC Cells

The HTonEpiCs were cultured and maintained according to the manufacturer’s guidelines. Briefly, HTonEpiCs were cultured in a PLL-coated flask (2 μg/cm^2^ T-75 flask) with complete growth medium (CGM) and incubated at 37 °C in a 5% CO_2_ humidified atmosphere. CGM was the mixture of growth supplement, penicillin/streptomycin solution, and tonsil epithelial cell medium [1:1:100 (*v*/*v*/*v*)]. Cells (approximately after 3–4 days of growth) were used for the in vitro assays and consistently showed a 95–100% viability, as determined by trypan blue staining. Cells were recovered from the flask using TE solution and then neutralized with TNS and washed with PBS prior to use.

### 2.3. Cell Viability Assay

#### 2.3.1. Spectrophotometric MTS Cell Viability Assay

Cell viability was measured using a standard MTS assay. Briefly, HTonEpiC cells were seeded at 10,000 cells/well in PLL coated 96-well plates and incubated for 24 h. The cells were washed with PBS and fresh CGM (100 µL/well) containing each treatment, carvacrol (4, 8, 16.1, 31.2, 62.5, 125, and 250 µg/mL), nimesulide (a positive control at 4, 8, 16 µg/mL), and LTA + PGA (5 µg/mL, each), or DMSO (a negative control, 0.05%, *v*/*v*), were added. Then, the plates were incubated for 24 h (5% CO_2_ at 37 °C). After 24 h of incubation, the media were discarded, and fresh CGM was added (100 µL/well). Then, 10 μL of MTS reagent (MTS: PMS (20:1)) was added to each well, and the plates were incubated at 37 °C for 2.5 h. The absorbances were measured at 490 nm using a microplate reader (Tecan Infinite™ M200 PRO, Tecan US Inc., Morrisville, NC, USA). The percentage of cell viability is calculated as (absorbance of treated well/absorbance of control well) × 100.

#### 2.3.2. Flow Cytometric Cell Viability Using the 7-AAD Assay

The HTonEpiCs were seeded at 200,000 cells/well in PLL-coated six-well plates at 37 °C. The next day, media was discarded, cells were washed with PBS, and CGM (1 mL/well) was added. Then, cells were incubated in the absence or presence of each treatment as carvacrol (8–250 µg/mL), nimesulide (4, 8, 16.1 µg/mL), LTA + PGN mixture (5 µg/mL, each), and DMSO (0.05%, *v*/*v*) for 24 h. Afterwards, 5 µL of lysis buffer (9% Triton X-100) (positive control) was treated for 5 min before the cell separation. Then, the cell monolayer was washed with PBS and detached by incubation with TE solution (1 mL/well) for 3 min. The cell suspension was neutralized with TNS (1.5–2 mL/well), and the cells were harvested by centrifugation (1000× *g*, 5 min). The cells were resuspended in PBS (300 µL/pellet) and incubated with 5 µL of 7-AAD (1 mg/mL) at room temperature (RT) for 5 min. Effects of carvacrol on cell viability (proliferation) were analyzed by flow cytometry using an Attune™ NxT acoustic focusing flow cytometer (AFC2, Thermo Fisher Scientific Inc., San Jose, CA, USA) and Attune™ NxT flow cytometry analysis software (v3.1.1234.0., Thermo-Fisher Scientific Inc., San Jose, CA, USA).

### 2.4. HTonEpiCs Inflammatory Cell Model

**Figure 2:**
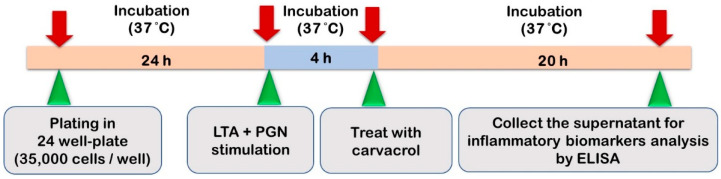
The LTA- and PGN-stimulated tonsil epithelial cell inflammatory model system used in the study.

The LTA- and PGN-stimulated tonsil epithelial cell inflammatory model system used in the study is shown in Figure 2. HTonEpiCs were cultured (density of 35,000 cells/well) in PLL-coated 24-well plates for 24 h. The cells were stimulated with a mixture of LTA and PGN (each at 10 μg/mL) at 37 °C. After 4 h, the cells were exposed to carvacrol (4–125 µg/mL) and nimesulide (4 µg/mL) for 20 h. DMSO (0.05%, *v*/*v*) was included as the experimental control. After incubation, the cultures were centrifuged (1000× *g*, 10 min), and the supernatants were saved at −80 °C for the determination of pro-inflammatory biomarkers. The concentrations of pro-inflammatory cytokines were measured using ELISA kits according to the manufacturer’s instructions.

### 2.5. Pro-Inflammatory Biomarker ELISA

The concentrations of pro-inflammatory cytokines were measured using ELISA kits according to the manufacturer’s instructions.

#### 2.5.1. IL-6 Assay

According to the manufacturer’s instructions, the protein concentration of IL-6 was measured by the ELISA kit supplied by BD Biosciences (Mississauga, ON, Canada). Briefly, 96-well Nunc-Immuno™ polystyrene Maxisorp ELISA flat-bottom plates (Thermo-Fisher Scientific Inc., Nepean, ON, Canada) were coated with anti-human IL-6 monoclonal antibody (100 µL/well) and incubated overnight at 4 °C. Uncoated spaces were blocked using assay diluent (1 h, RT), and wells were washed. Then, 100 µL of each standard (4.7–200 pg/mL), samples (2–250 µg/mL concentrations of carvacrol-treated cell supernatants), and controls were pipetted into wells. Plates were sealed and incubated for 2 h at RT with gentle shaking. Then, five well-washing steps were conducted, and 100 μL of the working detector (detection antibody + streptavidin-hoarse reddish peroxidase (HRP)) was added to each well. The covered plates were incubated for 1 h at RT. Following another seven washing steps, 100 μL/well of 3, 3’, 5, 5’-tetramethylbenzidine (TMB) substrate solution was added and incubated for 30 min at room temperature in the dark (without the sealer). Then, the stop solution was added (50 μL/well), and the absorbance readings were obtained at 450 nm. The concentration of IL-6 levels was calculated using a standard curve and expressed as a percentage of IL-6 concentration on the 24 h model.

#### 2.5.2. IL-8 Assay

The levels of IL-8 were determined by using the IL-8 ELISA kit according to the manufacturer’s guidelines. Briefly, 50 μL of each 2-fold diluted standard (0–1000 pg/mL), carvacrol (2–250 µg/mL), LTA + PGN (4 and 24 h), nimesulide (4, 8, and 16 µg/mL), and DMSO control were added, and 50 µL of human IL-8 biotin conjugate solution was also added. The plates were incubated for 1.5 h at RT with gentle shaking. After the supernatant was discarded, the plates were washed (4 times) after discarding the supernatant. Followed by four washing steps, streptavidin–HRP solution (100 μL per well) was added to each well except for the chromogen blanks, which were incubated (30 min, RT) with gentle shaking. The wells were thoroughly aspirated and washed four times, and subsequently, 100 μL of TMB substrate reagent was added to each well and incubated for 30 min at RT with gentle shaking in the dark. Then, 100 µL stop solution per well was added to stop the reaction. The solution in the well was changed from blue to yellow, which was measured at an absorbance of 450 nm. The IL-8 concentration was calculated using a standard curve, and the data were expressed as percentage of ENA-78 production.

#### 2.5.3. GCP-2 Assay

The assay was performed according to the manufacturer’s instructions. In the assay, 100 μL of each standard (0, 2, 500 pg/mL), carvacrol (2–250 µg/mL), LTA + PGN controls (4 and 24 h), nimesulide (4–16 µg/mL) and vehicle control were pipetted into the appropriate wells. Plates were incubated for 2.5 h at RT with gentle shaking. Then, the solution was discarded and washed with wash buffer. Then, a 100 µL of biotinylated antibody was added to each well and incubated for 1 h at room temperature with gentle shaking. This was followed by another washing step, and streptavidin solution (100 µL/well) was added. After incubation (45 min, RT) with gentle shaking, the wells were washed, and a 100 μL of TMB One-Step Substrate Reagent (TMB) was added into each well. The planes were covered and incubated for 30 min at RT in the dark. The absorbance at 450 nm was immediately measured after stopping the reaction by adding 50 μL of the stop solution. The protein concentration was calculated using a standard curve, and the data were expressed as percentage of GCP-2 concentration.

#### 2.5.4. ENA-78 Assay

The human ENA-78 ELISA kit was used to measure the protein production of ENA-78 according to the instructions provided by the manufacturer. The 96-well plates coated with anti-human ENA-78 were used, and the assay procedure was similar to the description in the GCP-2 assay in Section 2.5.3.

#### 2.5.5. TNF-α Assay

The TNF-α secretion in the cell culture supernatant was measured according to the manufacturer’s guidelines. Briefly, a 50 µL of ELISA diluent was pipetted into each well, and then, 100 µL of six standards (7.8–250 pg/mL), samples, and controls were added into the wells. The plates were sealed and incubated for 2 h at room temperature with gentle shaking. After the wells were aspirated and washed (3 times), 100 μL of the working detector (enzyme concentrate: detection antibody = 1:250) was added. Then, the plates were incubated (1 h, RT), aspirated, and washed (7 times). Then, 100 μL of substrate reagent, TMB, was added and further incubated for 30 min at RT in the dark. This was followed by adding 50 μL of the stop solution to each well, and the absorbance was read at 450 nm. The TNF-α concentrations were calculated using a standard curve and expressed as pg/mL.

#### 2.5.6. HBD-2 Assay

The concentration of HBD-2 was measured according to the guidelines provided by the HBD-2 ELISA kit. Briefly, anti-HBD-2 antibody-coated 96-well plates (Nunc-Immuno™ polystyrene Maxisorp, Thermo-Fisher Scientific Inc., Nepean ON, Canada) were prepared using capture antibody (0.5 µg/mL) incubation and then block buffer incubation according to the manufacturer’s guidelines. First, 100 μL of standards (0–2 ng/mL), carvacrol, or controls were added to each well and allowed to incubate for 2 h (RT). Next, the wells were washed four times with washing buffer and incubated for another 2 h (RT) with the detection antibody (0.5 µg/mL). After aspiration and washing, 100 μL of the avidin–HRP conjugate was added to each well and incubated for 30 min (RT). Then, 2, 2′-azino-bis (3-ethyl benzothiazoline-6-sulfonic acid) (ABTS) solution was added to the washed and dried wells. After the development of color, the absorbance of the wells was read at 405 nm. A standard curve was used to calculate the HBD-2 concentrations.

#### 2.5.7. PGE_2_ Assay

The PGE_2_ levels in the cell supernatants were measured using the human PGE_2_ ELISA kit according to the protocols and plate plan provided. Briefly, 100 µL of each diluted standards (0–4 ng/mL) and samples were added to the appropriate wells. Then, 50 µL of PGE_2_-alkaline phosphate (PGE2-ACE) tracer and 50 µL of PGE_2_-monoclonal antibody was added into the wells as described in the assay protocol provided by the manufacturer. The blanks, the total activity, the non-specific-binding, and the maximum binding wells were maintained. The plate was covered and incubated for 2 h at RT with orbital shaking. The wells were thoroughly aspirated and were washed (5 times) with wash buffer. Then, 200 µL of pNPP solution (5 nNPP tablets in 25 mL diethanolamine [DEA] buffer) was added to the wells, and the covered plates were incubated for 1 h at RT in the dark. Then, the absorbance was measured between 405 and 420 nm. The PGE_2_ concentration in each sample was calculated using the standard curves and equations provided in the kit protocol.

#### 2.5.8. COX-2 Assay

The COX-2 protein concentration of in cell culture supernatant was measured using the human COX-2 ELISA kit (Ray Biotech, Inc., Norcross, GA, USA) as described in the manufacturer’s assay protocol. Briefly, the standards (0–300 ng/mL), carvacrol (2–250 µg/mL), and controls were added into the appropriate wells. The plate was sealed and was incubated for 2.5 h at RT. The wells were emptied and washed using wash buffer seven times. Then, 100 µL of biotin antibody solution was pipetted into each well and incubated for another 1 h at RT. The wells were aspirated, washed (7 times), and 100 µL of streptavidin solution was added. After an incubation period of 45 min at RT, the wells were rewashed four times, and 100 µL of TMB substrate was added to each well. The reaction was facilitated for 30 min incubation at RT in the dark and was stopped by adding 100 μL of stop solution. The absorbance was read at 450 nm immediately, and the concentrations of COX-2 concentration were calculated using a standard curve and expressed in ng/mL.

### 2.6. Morphology Assessment of Cells in the Inflammatory Model

Cell morphology was examined under an inverted microscope (ECLIPSE TS 100/TS 100-F, Nikon Instruments Inc., Melville, NY, USA) with 400 X magnification. The images were captured using a Lumenara infinity camera (1-2 USB, 2.9 Megapixel) and processed using NIS elements software (v4.0, Lumenara Corporation, Ottawa, ON, Canada).

### 2.7. Statistical Analysis

The experiments were designed using a completely randomized design. All the experiments were conducted in triplicates (experimental triplicates) and repeated three independent times on different days and using different cell passages for a total of three biological replicates. Results were expressed as a mean ± standard error of the mean. Statistical analysis and designs of the graphs/figures were performed using Graph-prism™ software (v5.0, Informer Technologies, Inc., Los Angeles, CA, USA). One-way analysis of variance was used to determine the significant differences between means of pairs, which were resolved by using Tukey’s tests at *p* < 0.05, *p* < 0.01, and *p* < 0.001.

## 3. Results

### 3.1. Effect of Carvacrol on Cell Viability and Morphological Changes of Human Tonsil Epithelial Cells

**Figure 3:**
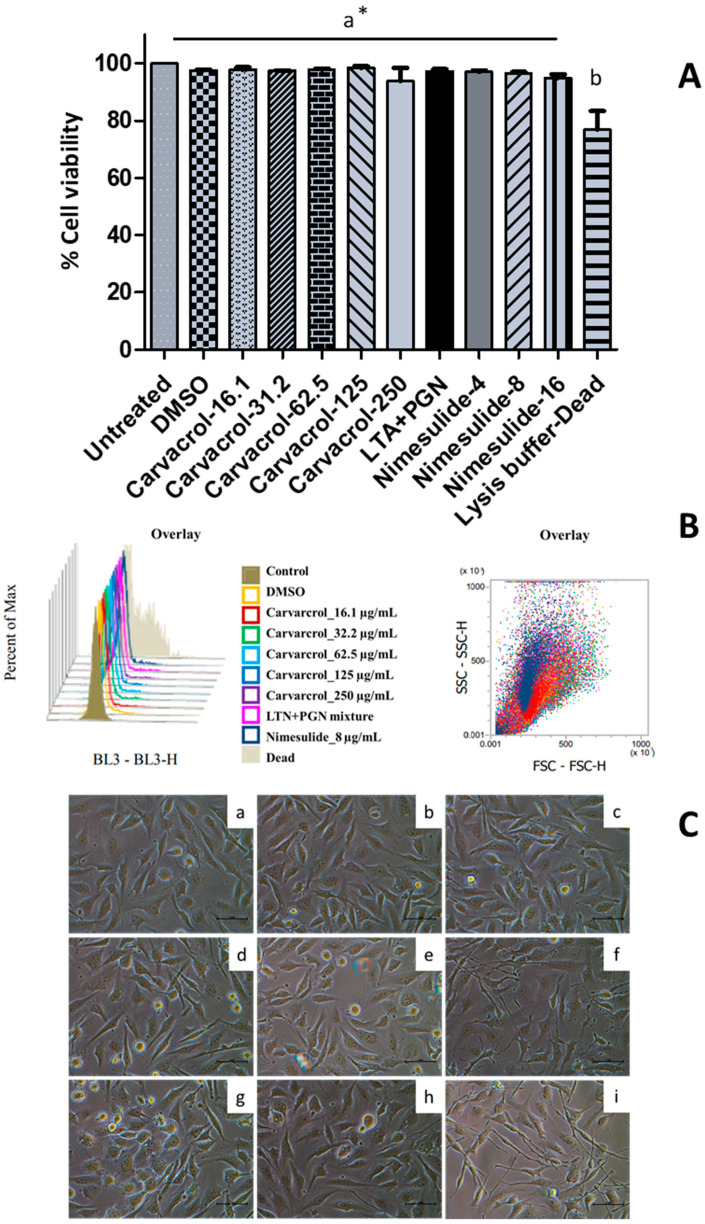
The effect of carvacrol on the viability of human tonsil epithelium cells; (**A**) Cell viability changes with 16.1, 31.5, 62.5, 125, and 250 µg/mL of carvacrol in DMSO, 4, 8, and 16.1 µg/mL of nimesulide, lipoteichoic acid (LTA) + peptidoglycan (PGN) (10 µg/mL each), and 0.05% dimethyl sulfoxide (DMSO) for 24 h were determined using 7-AAD staining followed by flow cytometry (FCM) analysis of human tonsil epithelial cells (HTonEpiCs). Absorbance was measured at 488 nm; Cell viability (%) was calculated relative to the control of 0.05% DMSO; Values are shown as mean ± SE from three independent experiments, each in triplicate; *, The different letters above the columns show that the means of different groups were significantly different (*p* < 0.05) by one-way analysis of variance using Tukey’s test; (**B**) The overlay of histograms and scatter plots of the controls and samples given by FCM analysis. (**C**) The morphological changes of the HTonEpiCs cells after the treatments were examined under an inverted microscope at 10 × 40 magnification; Representative photographs that were taken 24 h after the treatment in three independent experiments are presented; (**a**) untreated; (**b**) vehicle control (0.25% DMSO); and (**c**) 16 µg/mL carvacrol; (**d**) 31.5 µg/mL carvacrol; (**e**) 62.5 µg/mL carvacrol; (**f**) 125 µg/mL carvacrol; (**g**) 250 µg/mL carvacrol; (**h**) LTA + PGN (10 µg/mL, each); and (**i**) 8 µg/mL nimesulide. Scale bar = 1 mm. 7-AAD: 7-amino-actinomycin D; DMSO: dimethyl sulfoxide; LTA: lipoteichoic acid; and PGN: peptidoglycan.

We have recently demonstrated that carvacrol is not cytotoxic (3.9–250 µg/mL) to HTonEpiCs using the MTS assay [17]. To further assess whether carvacrol has a harmful effect on HTonEpiCs, the cells were incubated with carvacrol (8–250 µg/mL) or controls (DMSO as vehicle control, nimesulide as positive control) and the cell viability was analyzed by flow cytometry. The results showed that carvacrol at the tested concentrations was not cytotoxic (>80% cell viability) to HTonEpiCs by flow cytometric cell viability assay (Figure 3A,B). A set of representative histograms and scatter plots of the controls and carvacrol-treated samples are shown in Figure S1. In addition, cells treated with carvacrol did not show significant morphological changes compared to those treated with DMSO (Figure 3C). In addition, nimesulide, a known anti-inflammatory agent, showed no cytotoxicity to the cells at all the concentrations tested. Therefore, the concentrations of 16.1, 31.2, 62.5, 125, and 250 µg/mL carvacrol (in DMSO) and 4 µg/mL of nimesulide were used in the subsequent inflammation experimentation.

### 3.2. Effect of Carvacrol on Morphological Changes of the HTonEpiCs Inflammation Model

**Figure 4:**
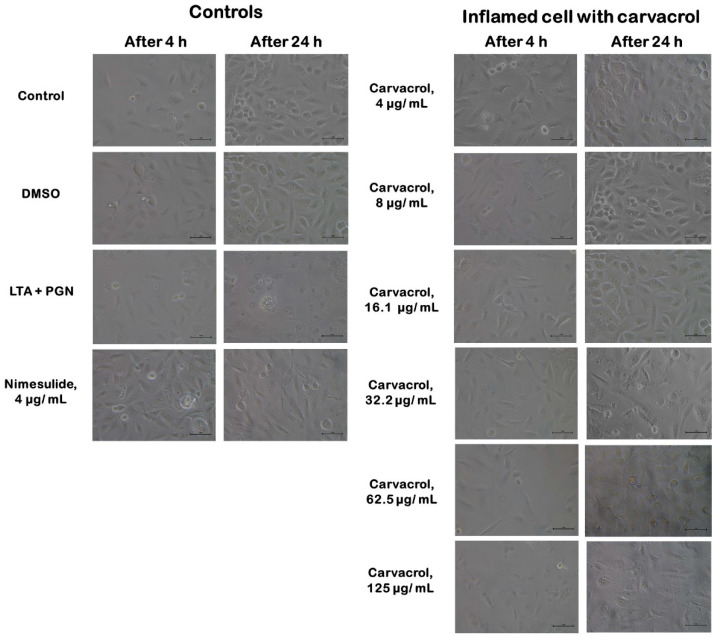
Morphology of human tonsil epithelial cells treated with carvacrol. Controls include cells of untreated growth medium control, DMSO control, LTA + PGN control, and nimesulide (4 µg/mL). LTA+PGN-induced cells (4 h) were treated with carvacrol (4–125 µg/mL) and incubated for an additional 20 hr. All images were obtained at a magnification of 10 × 40: scale bar = 1 μm. LTA: lipoteichoic acid; PGN: peptidoglycan; ELISA: enzyme-linked immunosorbent assay.

No changes in cell morphology or density were observed in untreated and vehicle (DMSO) controls-treated HTonEpiCs. However, after the 4 h incubation with LTA + PGN, some cells in the population showed morphological changes such as irregularity of shape, reduced size, and reduced cell density (Figure 4). The morphological changes were obvious after 24 h incubation with LTA + PGN.

### 3.3. Carvacrol Inhibits Pro-Inflammatory Cytokine Production by LTA + PGN-Stimulated HTonEpiCs

**Figure 5:**
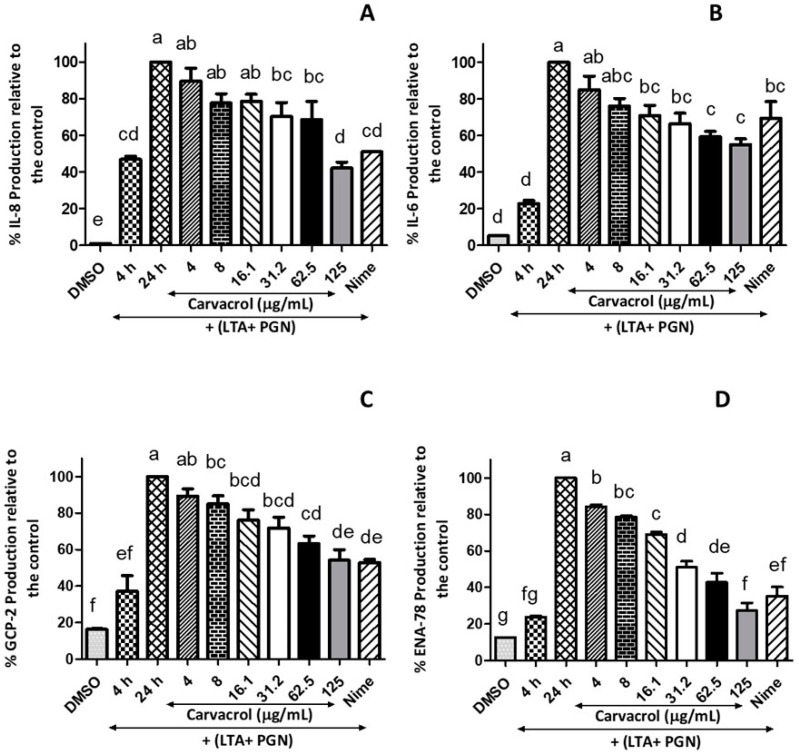
Inhibitory effects of carvacrol on the production of pro-inflammatory cytokines by LTA + PGN-stimulated human tonsil epithelial cells; Tonsil cells were treated with carvacrol (4–125 μg/mL) for 20 h after 4 h stimulation with LTA + PGN mixture (5 μg/mL, each). Supernatants were harvested 20 h after stimulation. Concentrations of (**A**) IL-8, (**B**) IL-6, (**C**) CGP-2, and (**D**) ENA-78 in the culture supernatants were determined by ELISA. Results are shown as the mean ± SE. Data are representative of three independent experiments conducted in triplicate. The different letters above the columns (a–g) show that the means of different groups were significantly different (*p* < 0.05) by one-way analysis of variance. Nime: nimesulide; IL-8: interleukin-8; IL-6: interleukin-6; ENA-78: epithelial-derived neutrophil-activating protein-78; GCP-2: granulocyte chemotactic protein-2; LTA: lipoteichoic acid; and PGN: peptidoglycan.

HTonEpiCs secreted pro-inflammatory cytokines, IL-6, IL-8, TNF-α, ENA-78, and GCP-2, when stimulated with LTA + PGN. In the absence of carvacrol, IL-6 and IL-8 at the 4 h (baseline level) stimulation were 24 and 35 pg/mL, and at the 24th h were 106 and 78 pg/mL, respectively. However, the levels of IL-6, IL-8, ENA-78, and GCP-2 were significantly decreased when the cells were treated with carvacrol in a concentration-dependent manner (Figure 5). It was observed that there was a decrease of IL-6 and IL-8 to 55% and 42% (*p* < 0.0001), respectively, in this cell model with the presence of 125 µg/mL carvacrol. Interestingly, at higher concentrations of carvacrol (125 and 62.5 µg/mL), the suppression of pro-inflammatory cytokines is similar to that of nimesulide (4 µg/mL) (*p* < 0.05).

Significant concentration-dependent suppression of GCP-2 (also known as chemokine ligand 6, CXCL6) production by carvacrol compared to the antigen control was observed (Figure 5C). Carvacrol at 62.5 and 125 µg/mL showed a 63.2% and 51.1%, respectively, reduction in GCP-2 production compared to the control, which is a level similar to 4 µg/mL of nimesulide. In addition, the production of ENA-78 was suppressed by 3.36-fold when the cells were treated with 125 µg/mL of carvacrol compared to the 24 h-model, whereas nimesulide (4 µg/mL) showed only a 2.8-fold reduction of the ENA-78 (Figure 5D). However, the analysis indicated that carvacrol did not affect the secretion of TNF-α (data are not presented).

### 3.4. Inhibitory Effects of Carvacrol on the Secretion of HBD-2, COX-2, and PGE_2_

**Figure 6:**
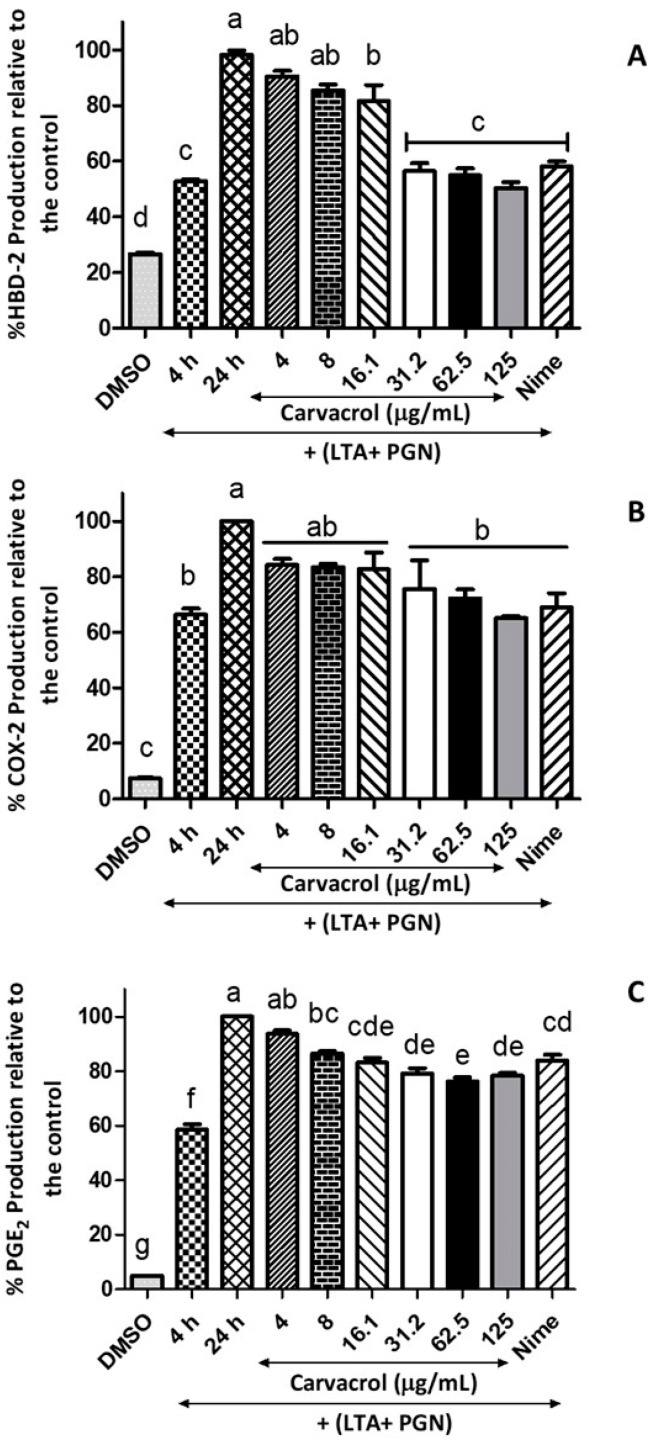
Inhibitory effects of carvacrol on the production of HBD-2, COX-2, and PGE_2_ in LTA- and PGN-stimulated human tonsil epithelial cells; Tonsil cells were treated with carvacrol (4–125 μg/mL) for 20 h after 4 h stimulation with an LTA and PGN mixture (5 μg/mL, each). Supernatants were harvested 20 h after stimulation. Production of (**A**) HBD-2, (**B**) COX-2, and (**C**) PGE_2_ in the culture supernatants were determined by ELISA. Results are shown as the mean ± SE. Data are representative of three independent experiments conducted in triplicate. The different letters above the columns (a–g) show that the means of different groups were significantly different (*p* < 0.05) by one-way analysis of variance using Tukey’s test. HBD-2: human beta defensin-2; COX-2: cyclooxygenase 2; PGE_2_: prostaglandin E2; LTA: lipoteichoic acid; and PGN: peptidoglycan.

The effects of carvacrol on modulating the anti-microbial peptide HBD-2 production by HTonEpics were assessed. A decrease in the production of HBD-2 in a carvacrol-concentration-dependent manner was observed in comparison to the 24 h-model control (Figure 6). Furthermore, the anti-bacterial minimum inhibitory concentration (MIC) of carvacrol (125 μg/mL) reduced the release of HBD-2 by over 50%, while the positive control of nimesulide (4 μg/mL) also showed a similar reduction.

Next, we investigated the effects of carvacrol on COX-2 and PGE_2_ productions by LTA- and PGN-stimulated HTonEpiCs. After a 24 h incubation with LTA and PGN, the cells produced an increased level of COX-2 and PGE_2_ compared to the control (*p* < 0.05) (Figure 6). Interestingly, when LTA- and PGN-stimulated cells were incubated with carvacrol, significantly decreased levels of COX-2 (at and above 31.5 μg/mL carvacrol) and PGE_2_ (at and above 8 μg/mL carvacrol) were observed. The effects of carvacrol on COX-2 and PGE_2_ production were concentration-dependent.

## 4. Discussion

The use of phytochemicals, herbal extracts, or herbal formulas of traditional medicine has been reported to manage inflammatory diseases, including those associated with the respiratory tract [9,18]. The anti-inflammatory activities of carvacrol have been demonstrated in various cells such as RAW 264.7 macrophages [12,19], and animal models, such as paw inflammation in mice [16] and colitis-associated colon cancer of male Fischer 344 rats [15]. Anti-inflammatory activities of carvacrol were reported along with the suppression of pro-inflammatory cytokines. The suppression of interleukin IL-8 and HBD-2 secretion in LPS-stimulated airway epithelial A549 cells by an herbal formulation against respiratory infections was reported [18]. However, the anti-inflammatory activity of carvacrol in the upper respiratory tract, particularly when the inflammation is a result of an *S. pyogenes* infection, has not been reported.

In this study, a mixture of LTA and PGN, two Gram-positive bacterial antigens, were used to stimulate HTonEpiCs, which represented an in vitro model of streptococcal pharyngitis. Tonsil inflammation is initiated by complex processes that are triggered by streptococcal cell wall antigens such as LTA and PGN. When bacteria colonize the tonsil, various innate immunity-associated cells, including pharyngeal and tonsillar epithelial cells, neutrophils, and macrophages [20], begin to secrete inflammatory mediators, such as cytokines and chemokines, anti-microbial peptides (AMPs), and eicosanoids, including PGE_2_ [4]. After exposure to *S. pyogenes* or its cell wall components, the epithelial cells and macrophages act as the first line of defense [4]. Other inflammatory cells migrate to the site of inflammation at a later stage.

**Figure 7:**
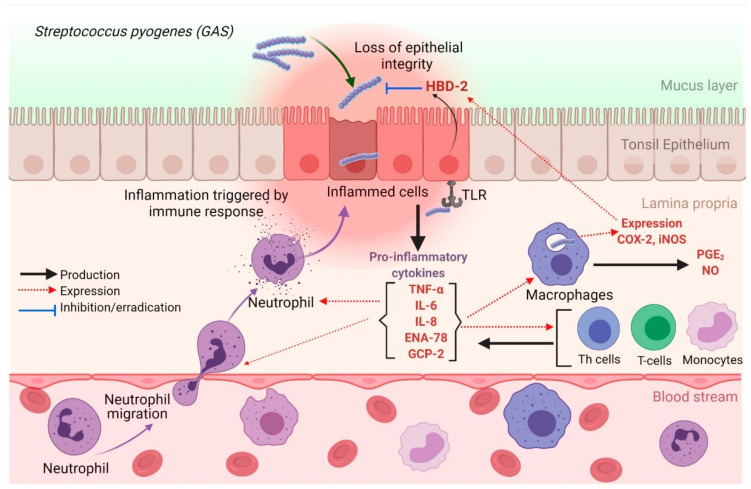
Possible mechanisms of releasing pro-inflammatory cytokines by tonsil epithelial cells during GAS infection. TLRs recognize GAS during the invasion, which results in the release of inflammatory cytokines by inflamed epithelial cells. The secretion of pro-inflammatory cytokines (IL-6 and IL-8) potently promotes the recruitment of neutrophils and macrophages to the site of inflammation. TNF-α activates the NF-κB pathway, which facilitates the expression of pro-inflammatory and cell survival genes. GAS: Group A streptococcus; IL-6: interleukin-6; IL-8: interleukin-8; iNOS: inducible nitric oxide synthase; HBD-2: human beta defensin-2; GCP-2: granulocyte chemotactic protein-2; NF-κB: nuclear factor-kappa B; ENA-78: epithelial-derived neutrophil-activating protein-78; NO: nitric oxide; TLR: Toll-like receptors, TNF-α: tumor necrotic factor-alpha. The figure was created using BioRender.com.

*S. pyogenes* adheres to tonsillar epithelial cells during infection, triggering these cells to release cytokines (Figure 7). Our results demonstrated that tonsil epithelial cells produced a spectrum of inflammatory biomarkers upon stimulation by LTA and PGN. Furthermore, we showed that carvacrol could suppress the production of pro-inflammatory cytokines and could theoretically reduce inflammation. Our results are in agreement with previous studies demonstrating the anti-inflammatory activity of carvacrol, its derivatives, or carvacrol-rich plant extracts that inhibit the production of pro-inflammatory cytokines and inflammatory mediators such as COX-2, PGE2, nitric oxide (NO), and inducible nitric oxide synthase (iNOS) during respiratory tract inflammations [9,14,21,22].

The significance of Gram-positive bacteria in the induction of IL-6 and IL-8 release was studied previously, and dose-dependent IL-6 and IL-8 release were demonstrated from both macrophages and respiratory epithelial cells after stimulation with bacteria [23]. IL-6 is primarily a pro-inflammatory cytokine-mediated by trans-signaling during an infection; it is also responsible for many immunoregulatory and anti-inflammatory activities mediated by classical signaling [24]. In addition, IL-8 is a chemotactic agent (chemokine), facilitating the migration and recruitment of neutrophils and T-cells and priming eosinophils [25]. A recent meta-study reported a positive effect of carvacrol on the reduction of IL-1β, IL-4, and IL-8; however, there was no effect on IL-6 and TNF-α, which was probably due to the methodological quality and heterogeneity of the studies [26]. A recent study found that lipopolysaccharide (LPS) stimulated J774.1 mouse macrophages expressed the genes of IL-1β and TNF-α and carvacrol and thymol could significantly reduce the production of both IL-1β and TNF-α at the protein and mRNA levels [27]. However, we did not detect TNF-α in our experiments. This could be because HTonEpiCs are not being specialized to produce TNF-α or the antigen concentration used was not high enough to generate detectable levels of TNF-α.

The innate immune system of the host provides the first line of defense against bacterial infection. Host cells recognize pathogen-associated molecular patterns (PAMPs) through pattern-recognition receptors (PRRs), such as TLRs [28]. Among these TLRs, TLR2 recognizes LTA and PGN, whereas TLR4 recognizes LPS [28]. Respiratory tract epithelial cells express anti-microbial peptides such as HBDs to inhibit bacterial proliferation during infections, and they are important to innate host defenses [29,30]. In general, HBD-2 is upregulated in epithelial cells and mononuclear phagocytes in response to bacterial infection and associated pro-inflammatory cytokine release [31]. The upregulation of HBD-2 by cell wall components such as LTA and PGN in Gram-positive bacteria [32] and LPS in Gram-negative bacteria [31] has been reported. The present study confirmed this phenomenon that an antigen mixture of LTA and PGN induces HTonEpiCs and increases HBD-2 production. Interestingly, a concentration-dependent reduction of HBD-2 production was observed in carvacrol-treated cells.

Our results further showed the suppression of PGE_2_ and COX-2 production, which are also significant mediators of inflammation. PGE_2_ plays a vital role in the inflammatory processes of pharyngitis and is synthesized from arachidonic acid through the COX-1 and COX-2-involved biosynthesis of eicosanoids [7]. Therefore, blocking or inhibiting the COX-2 enzyme could potentially decrease PGE_2_ production and thus reduce pharyngeal inflammation. COX-2 inhibitors suppress PGE_2_ production, which ultimately reduces inflammation [7]. Several studies have proposed the role of the PGE_2_ during streptococcal pharyngitis [7]. Landa et al. suggested that the effect of carvacrol is mediated by inhibiting COX-2, which decreases PGE_2_ production [22].

Many of these inflammatory markers act interconnectedly during inflammation (Figure 7). It was reported that PGE_2_ and COX-2 increase IL-8 expression in human pulmonary epithelial cells during inflammation [33]. Our study demonstrated that carvacrol suppressed PGE_2_, COX-2, and IL-8 production. Furthermore, neutrophil chemotactic chemokines such as GCP-2 and ENA-78 may play a role in IL-8 production. According to Frick et al. [34], the cells that produce IL-8 have also affected the secretion of ENA-78 and GCP-2. Our results also showed that IL-8 has a similar secretion pattern to ENA-78 and GCP-2 by the tonsil epithelial cells after antigen stimulation. There are several categories of chemokines based on the position of conserved cysteine residues where GCP-2, ENA-78, and IL-8 are considered C-X-C chemokines (CXC) containing one amino acid between the two NH_2_-terminal cysteine residues [35]. GCP-2 (CXCL6) is more structurally related to ENA-78 (CXCL); however, it functionally utilizes both of the IL-8 (CXCL8) receptors to chemoattract neutrophils [36]. The relationship between LPS-induced GCP-2 and IL-8 production in fibroblasts was studied [36], and our current study showed the interconnected production of GCP-2, ENA-78, and IL-8 in LTA- and PGN-stimulated HTonEpiCs. Carvacrol treatment suppressed the production of all three of the cytokines mentioned above, suggesting the potential of carvacrol in inflammation reduction.

Based on the previously reported in vitro and in vivo studies, carvacrol exerted anti-inflammatory effects at one or multiple cellular targets. It has been suggested that an interaction of transcription factors such as nuclear factor kappa-light-chain-enhancer of activated B cells (NF-κB) and pro-inflammatory mediators such as IL-6, TNF-α, and COX-2 during inflammation is a major concern [37]. Furthermore, it has been reported that carvacrol blocks the NF-κB nuclear translocation and transcriptional activation in LPS-induced pro-inflammatory activation in RAW 264.7 macrophages [12]. Interestingly, a few other recent studies have also reported that carvacrol and carvacrol- rich plant extracts ameliorate the suppression of NF-κB signaling pathway and thereby downregulate the pro-inflammatory genes such as iNOS, IL-6, IL- 1β, COX-2, and TNF-α under various inflammatory conditions [14,27,37]. Therefore, we suggest that the anti-inflammatory activity of carvacrol on HTonEpiCs could also be due to the suppression of NF-κB signaling pathways. Further studies are required to assess the precise mode of action of carvacrol against HTonEpiCs inflammation induced by Gram-positive bacterial antigens.

Our findings indicate that carvacrol-containing lozenges or throat sprays for pharyngitis patients can be developed. Previously, we have found that carvacrol has rapid anti-streptococcal activity [17], and here, we provide evidence that carvacrol also possesses anti-inflammatory properties. More importantly, carvacrol is not cytotoxic to human epithelial cells and has been approved by the Food and Drug Administration (FDA) for use in foods (Code for Federal Regulation: 21CFR172.515). Therefore, carvacrol could potentially be an excellent candidate for an oral throat pain-relieving natural health product.

## 5. Conclusions

In conclusion, the present study indicates that carvacrol suppresses the production of pro-inflammatory mediators such as IL-6, IL-8, HBD-2, GCP-2, ENA-78, PGE_2_, and COX-2, suggesting that it has anti-inflammatory properties. Therefore, further investigations can be continued to develop carvacrol as a natural health additive for inclusion in products such as lozenges or throat sprays for pain management associated with streptococcal pharyngitis.

## Data Availability

Data is contained within the article and Supplementary Materials.

## Supplementary Materials

The following are available online at https://www.mdpi.com/article/10.3390/nu14030503/s1, Figure S1: A set of representative histograms and scatter plots of the controls and carvacrol samples given by FCM analysis in human tonsil epithelial cells. Cell viability after 24 h treatment of controls (untreated, DMSO, Nimesulide, LTA + PGN mixture) and carvacrol (16.1, 31.2, 62.5, 125, and 250 µg/mL) was determined using 7-AAD staining for 5 min. Forward scattering (FSC) and side scattering (SSC) plots are also included.
