# Supplementary material for: Carvacrol Suppresses Inflammatory Biomarkers Production by Lipoteichoic Acid- and Peptidoglycan-Stimulated Human Tonsil Epithelial Cells
Source: Nutrients. 2022 Jan 24;14(3):503. doi: 10.3390/nu14030503 (PMC8840435; doi:10.3390/nu14030503)
Supplement: Supplementary file 1 [file nutrients-14-00503-s001.zip › nutrients-1506926-supplementary.pdf]

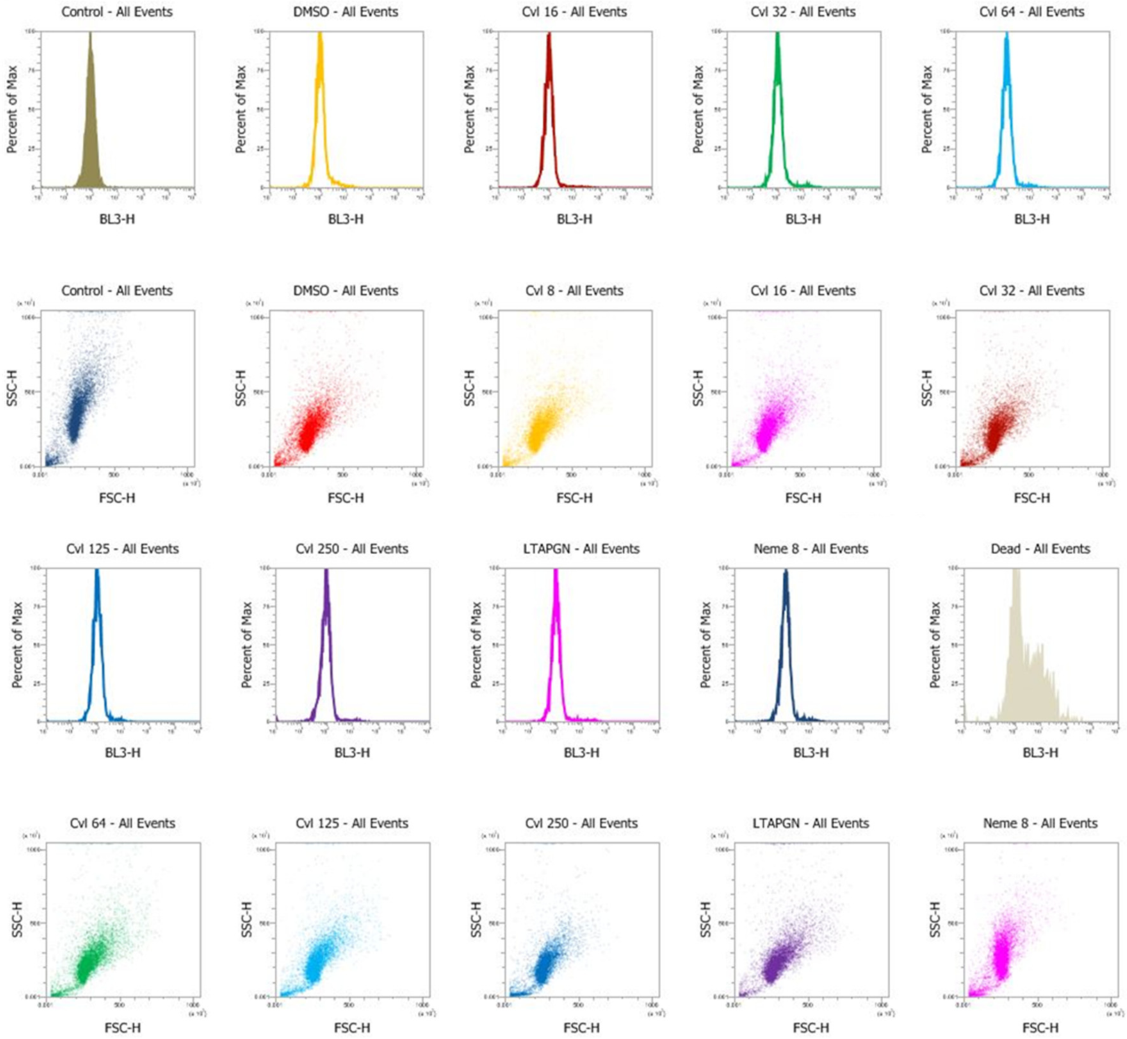

Figure S1. A set of representative histograms and scatter plots of the controls and carvacrol samples given by FCM analysis in human tonsil epithelial cells. Cell viability after 24 hr treatment of controls (untreated, DMSO, Nimesulide, LTA + PGN mixture) and carvacrol (16,32,64,125, and 250 μg/mL) was determined using 7-AAD staining for 5 min. Forward scattering (FSC) and side scattering (SSC) plots are also included.
